# Exploring the barriers and facilitators of psychological safety in primary care teams: a qualitative study

**DOI:** 10.1186/s12913-021-06232-7

**Published:** 2021-03-24

**Authors:** Ridhaa Remtulla, Arwa Hagana, Nour Houbby, Kajal Ruparell, Nivaran Aojula, Anannya Menon, Santhosh G. Thavarajasingam, Edgar Meyer

**Affiliations:** 1 College of Medical and Dental Sciences, University of Birmingham, Birmingham, UK; 2 Imperial College London, School of Medicine, London, UK; 3 Leeds University Business School, University of Leeds, Leeds, UK

**Keywords:** Psychological safety, Teamwork, Primary care, General practice, Community

## Abstract

**Background:**

Psychological safety is the concept by which individuals feel comfortable expressing themselves in a work environment, without fear of embarrassment or criticism from others. Psychological safety in healthcare is associated with improved patient safety outcomes, enhanced physician engagement and fostering a creative learning environment. Therefore, it is important to establish the key levers which can act as facilitators or barriers to establishing psychological safety. Existing literature on psychological safety in healthcare teams has focused on secondary care, primarily from an individual profession perspective. In light of the increased focus on multidisciplinary work in primary care and the need for team-based studies, given that psychological safety is a team-based construct, this study sought to investigate the facilitators and barriers to psychological safety in primary care multidisciplinary teams.

**Methods:**

A mono-method qualitative research design was chosen for this study. Healthcare professionals from four primary care teams (*n* = 20) were recruited using snowball sampling. Data collection was through semi-structured interviews. Thematic analysis was used to generate findings.

**Results:**

Three meta themes surfaced: shared beliefs, facilitators and barriers to psychological safety. The shared beliefs offered insights into the teams’ background functioning, providing important context to the facilitators and barriers of psychological safety specific to each team. Four barriers to psychological safety were identified: hierarchy, perceived lack of knowledge, personality and authoritarian leadership. Eight facilitators surfaced: leader and leader inclusiveness, open culture, vocal personality, support in silos, boundary spanner, chairing meetings, strong interpersonal relationships and small groups.

**Conclusion:**

This study emphasises that factors influencing psychological safety can be individualistic, team-based or organisational. Although previous literature has largely focused on the role of leaders in promoting psychological safety, safe environments can be created by all team members. Members can facilitate psychological safety in instances where positive leadership behaviours are lacking - for example, strengthening interpersonal relationships, finding support in silos or rotating the chairperson in team meetings. It is anticipated that these findings will encourage practices to reflect on their team dynamics and adopt strategies to ensure every member’s voice is heard.

**Supplementary Information:**

The online version contains supplementary material available at 10.1186/s12913-021-06232-7.

## Background

Psychological safety is the notion where individuals feel empowered to ask questions, admit mistakes or voice concerns without fear of negative repercussions from their team [1]. This concept has been explored in varying contexts, including healthcare teams as psychological safety can have an impact on patient safety and quality of care. For healthcare professionals, psychological safety creates an environment of trust and openness to discuss concerns and raise errors [2, 3]. This enables focus on providing high quality care, as opposed to managing the expectations around voicing dissent and disagreement. It has also been shown that psychological safety increases physician engagement [4], reduces burnout [5] and promotes creativity [6].

Appelbaum et al. surveyed 106 physicians in the United States in order to investigate the perceptions of psychological safety and various other parameters including the intention to report adverse events. Psychological safety was found to be a direct predictor of the intention to report adverse events by physicians, highlighting the importance of psychological safety in creating safer care for patients [7]. Yanchus et al. investigated 11,726 healthcare workers including psychiatrists and mental health nurses and determined that psychological safety was a direct predictor of turnover intent, emphasising the value of psychological safety in employee retention [8].

Indeed, the positive effects of psychological safety are not limited to the individual or team level - rather, they permeate throughout the entire organisational infrastructure. This draws on the concept of organisational resilience, which can be described as how well supported workers within an organisation are by across three specific levels: the individual level, team level, and organisational level [9]. Organisations which are resilient will facilitate workers to predict when a problem will arise (foresight), help individuals cope with problems which do occur (coping), and finally, find suitable ways to recover from problems and prevent them in the future (recovery) [9]. In turn, organisational resilience allows for problem management, which in a healthcare setting translates to improved patient safety measures – a typical example of organisational resilience in healthcare is the clinical handover which aims to facilitate foresight, coping and recovery across the three levels of an organisation [9]. Psychological safety is integral to maintaining organisational resilience. For example, an individual healthcare worker should feel able to raise a concern regarding a patient showing clinical signs of deteriorating (foresight) without fear of repercussions from seniors [9].

In light of the well-evidenced benefits of psychological safety on healthcare teams, it is imperative to understand the key drivers which either facilitate or act as a barrier to establishing psychological safety. Specific facilitators which have already been identified in the literature include those pertaining to the actions of leaders. For example, inclusive behaviours displayed by a leader such as active invitation and appreciation of opinions from fellow team members regardless of factors such as hierarchical differences between a leader and team member have been shown to facilitate psychological safety, exemplified by Hirak et al’s [10] study which investigated the correlation between leader inclusiveness and psychological safety within a hospital [3, 11]. 224 team members and 55 team leaders consisting of various hospital employees including doctors and nurses were surveyed, and a positive relationship was found to exist within teams with more inclusive leaders [10].

The literature also links psychological safety with change-oriented leadership. Change-oriented leadership as described by Yuki et al [12] refer to a set of behaviours which promote innovation and change amongst teams. For example, leaders who monitor the external environment to identify opportunities or potential threats to a team, envision change, encourage innovation from their subordinates and take on personal risk to enact change are seen to be change-oriented leaders. Ortega et al [2] surveyed 107 nursing teams from various healthcare settings including primary care, intensive care and surgical settings to investigate the relationship between psychological safety and change-oriented leadership. Ortega et al. reported that teams with change-oriented leaders also reported higher psychological safety within teams [2]. This has great implications for healthcare considering innovation and non-traditional problem-solving strategies have historically proved beneficial for the industry.

Ethical leaders i.e. individuals who demonstrate appropriate conduct themselves and by doing so encourage and model exemplary conduct in their subordinates have also been cited in the literature as encouraging psychological safety [13]. Gong et al [14] surveyed the opinions of feedback-seeking behaviour amongst subordinate nurses and nurse leaders – in total, 60 leaders and 458 subordinates were investigated. Teams, where leaders were deemed to be more ethical, were found to have higher levels of psychological safety and feedback-seeking behaviour, particularly in teams with a high-power distance [14].

Barriers to psychological safety include workplace bullying and hierarchy. Arnetz et al [15] investigated the experience of workplace bullying amongst 331 registered nurses from a specific American regional healthcare system. 36.9% of responders reported being bullied in the preceding 6 months [14]. An inverse relationship was found between personal experiences of disengagement with work following personal bullying and psychological safety. Psychological safety was also associated with less personal bullying as well as witnessing others being bullied [15]. Hierarchy has also been cited in the literature, with Appelbaum et al [7] investigating the influences of power distance and leader inclusiveness on psychological safety amongst 106 medical residents. A higher perceived power distance predicted lower levels of psychological safety, whilst leader inclusiveness was positively correlated with psychological safety [7]. Higher levels of psychological safety by consequence were positively correlated with intentions to report adverse medical events, further highlighting the importance of mitigating barriers to psychological safety in order to maintain and improve patient safety.

Whilst the literature makes clear that leaders are crucial in facilitating psychological safety in healthcare teams, there is less focus on how other team members may help to improve the psychological safety of their environment. Circumstances where individuals speak up regardless of the leadership style they work under, suggests that other factors external to the leader are at play in facilitating psychological safety. Given that the literature has a strong focus on the role of the leader, attempts should be made to determine if general team behaviours, environmental factors, team culture or innate personality traits contribute to the psychological safety of a team environment and if so, what these factors may be. Likewise, are there alternative intrinsic or extrinsic factors that individuals may possess which can facilitate or impede the establishment of a psychologically safe environment.

Most of these findings on psychological safety in healthcare teams however, focuses on secondary care, with limited studies examining the application of this construct within primary care teams [3, 11]. Arguably, the dynamics of teamwork can vary greatly between primary and secondary care multidisciplinary teams, thus a focused exploration into psychological safety in these teams is warranted.

This qualitative study aimed to identify the specific barriers and facilitators of psychological safety in primary care teams. In the context of this study, barriers and facilitators refer to the various psychological, environmental, interpersonal and organisational aspects of the multidisciplinary teams investigated. This was with a view to establish behaviours that practices can implement to harbour psychologically safe environments.

Given that the aim of this study is to identify barriers and facilitators of psychological safety within primary care teams, an inductive study approach was deemed to be a more suitable study design as opposed to a traditional hypothetico-deductive approach [16]. The lack of specific premises to prove or disprove in the context of psychological safety further supports the use of an inductive methodology [17].

## Methods

### Research philosophy and approach

This study utilised a mono-method qualitative research design which uses semi-structured interviews as the only mode of data collection. The present study seeks to investigate multi-disciplinary team members’ perceptions of the facilitators and barriers of PS in primary care teams. Such perspectives and insights can only be explored using a qualitative inquiry which, crucially, uses methods such as open-ended interviewing to surface opinions unconducive to quantification [18].

This study employed an interpretivist approach which leverages qualitative methods to elicit narratives, capture stories and probe perceptions to articulate and conceptualise aspects of social phenomena which cannot be quantified [19]. Interpretivism champions subjectivity, and calls on the researcher to engage their own values and beliefs, making their empathetic viewpoint a central part of the research process [20]. Critical to the interpretivist philosophy is its acknowledgement of multiple realities and therefore, this approach facilitates a deep understanding of participants’ lived experiences [21].

The very notion that within the same context there exist multiple realities experienced by different people makes an interpretivist approach appropriate for the present study exploring MDT members’ views on PS in primary care teams. By exploring PS through the lens of different MDT members, this research acknowledges the complexity of the social world and seeks to develop a deep understanding of the phenomenon under investigation.

This study applies an inductive approach to theory development, which recognises the existence of a gap between observed data and derived conclusions [22]; a gap filled with underlying complexities which cannot always be distilled to ‘cause and effect’ mechanisms [20]. Inductive reasoning therefore traverses the rigid structural boundaries which govern deductive approaches and does not seek to mechanistically verify or oppose existing theory. Rather, an inductive approach is limitless. It utilises a ‘bottom up approach’ beginning with primary data collection followed by the identification of patterns and themes in an effort to construct theory [23]. Consistent with an inductive approach, this study uses qualitative methods focussed on meaning-making, allowing for a detailed exploration of participants’ lived experiences [24].

Methodology is reported in accordance with the Consolidated Criteria for Reporting Qualitative Research Checklist [25].

### Sampling

Snowball sampling enabled the recruitment of a team-focused study population, thus facilitating comparison between the perceptions of different MDT members. This was vital given that psychological safety is a team construct. Utilising snowball sampling methodology, a sample of 20 individuals from four different primary care teams (*n* = 5, *n* = 6, n = 6, *n* = 3) were obtained. The sampling approach was employed in two stages. First-line participants were recruited through LinkedIn and the Royal Colleges, subject to specified inclusion and exclusion criteria (Table 1). These participants then recruited colleagues from their multidisciplinary team. For example, to recruit the participants in team 1, the head partner GP was contacted through LinkedIn. They then initiated contact with the head nurse from the team which resulted in a sample of five participants in team 1. Their employment information was verified at the time of the interview by asking their role in the practice. The response rate through LinkedIn was approximately 70% and recruitment was completed in one month. The inclusion/exclusion criteria were checked prior to the interview by asking preliminary questions to obtain their professional role. The roles included were general practitioners, practice managers, partners, healthcare assistants and nurses. The demographic information has been anonymised due to the inclusion of direct quotes being used in this report. All recruitment was in line with the approved ethics protocol. A brief synopsis outlining the study purpose and objectives were sent to the participants. Once interest was confirmed, they were provided with a participant information sheet detailing the purpose of the study and information regarding data confidentiality alongside an informed consent form to obtain consent prior to interview conduction. Participants were informed that they could withdraw from the study at any time. This was repeated until no further recruitment occurred [26] and data saturation was reached. Data saturation was deemed the point at which similar responses were being surfaced in the interviews with repeating rather than novel ideas, referred to by Sandelowski [27] as ‘informational redundancy’. In qualitative research, significant ambiguity exists around what is deemed an appropriate sample size [20] with limited guidance on this. Guest et al. 2006 suggest that 12 interviews are sufficient [28], while Creswell [29] recommends between 5 and 30 interviews for qualitative research. An accepted sample size of between 5 and 25 participants has been cited for studies utilising semi-structured or in-depth interviews [30]. Therefore, given the fact that data saturation was achieved at 20 interviews, this was deemed an appropriate sample size for the study.

**Table 1:** Inclusion and exclusion criteria for participant recruitment

Inclusion Criteria	Exclusion Criteria
Healthcare professionals working in primary care teams	Healthcare professionals working in secondary care teams
London primary care teams	Non-London primary care teams
English speaking	Non-English speaking

### Data collection

Data was collected using semi-structured interviews (SSIs), as they are adaptable in nature and allow stakeholders to share answers openly and independently [31]. Interviews with all 20 participants were conducted via video-conferencing (due to Covid-19 restrictions). Video conferencing platforms utilised included Zoom and Skype. Conducting the interviews in this manner offered numerous advantages including; convenience for both the interviewer and the interviewee as well as deducting travel time, thus increasing efficiency of data collection. Furthermore, this facilitates visual interaction with the added advantage that it allows the interviewer and interviewee to remain in their own comfortable locations [32]. However, video-conferencing limited our non-verbal communication which could have helped contextualise the responses. Overall, utilising video-conferencing proved advantageous in our data collection process. Interviews were audio-recorded, anonymised and stored on a secure drive before being destroyed post-transcription.

The interview schedule was designed to be open-ended to encourage participants to speak freely to allow detailed accounts to be elicited [33]. This was recommended by the five-step framework by Kallio et al [34] to create a qualitative interview guide. Kallio et al. recommended first to evaluate if a semi-structured interview is necessary. The conclusion of conducting interviews was reached as this study needed the perceptions and opinions of our participants in order to contextualise their answers. Next, a literature review was conducted to establish existing knowledge and identify the gap the interview needs to fill. This helped us with the third step of devising the questions, which included the main themes and follow up questions.

As per Kallio et al’s fourth step [34], two pilot interviews with GPs were conducted to verify the initial interview guide developed. The pilot interviews demonstrated significant overlap in the interview guide questions within the subsection “Roles and Responsibilities”, hence this subsection was summarised into three questions. Secondly, the question ‘How do you view your relationship with other team members? was removed since it required extensive clarification in both pilots. Finally, one question was added to the interview protocol, ‘Which member of the team is most influential in ensuring a psychologically safe environment?’, due to both interviewees referring frequently to the influential role of team leaders in facilitating PS within their teams. Yin [35] advocates the conduction of pilot studies as an effective method for developing ‘relevant lines of informed questioning’, enabling the refinement of data collection methods. The conduction of pilot interviews further informed the modification of the interview guide to ensure data gauged from the questions was sufficient for answering our research question.

The semi-structured interview format allowed for probing questions to be used to encourage participants to develop and elaborate on their responses, facilitating a more detailed inquiry [36]. All SSIs ranged from 20 to 45 min in duration due to differences in individual availability and commitment of the respondents. This is in line with accepted practice in the literature [37]. Three researchers (KR, NA and NH) conducted the interviews which introduced different perspectives who were able to individually interpret the participants’ non-verbal cues and the emotional aspects which often do not surface in the transcripts and are only picked up in the interview. The triangulation of researchers [38] in this manner minimised individual biases and contributed to the validity of our research. An interview schedule (Supplementary file A) was devised with open-ended questions to encourage participants to speak freely, facilitating a detailed inquiry [33].

### Data analysis

Braun and Clarke’s six-phase methodology [39] of thematic analysis was utilised for the interview data. Phase 1 involved three researchers (RR, NH and AH) transcribing the interviews *ad verbatim* and developing transcript summaries. In line with an inductive approach, within phase 2, ‘in-vivo’ codes were derived from the data. Codes were reviewed and compared at the team level in phase 3 and were subsequently categorised into themes, beginning the process of theory inception. In the fourth phase, candidate themes and subthemes were reviewed against the coded data to ensure intra-theme coherence and against the entire data to ensure representability. Further refinement of themes was undertaken in phase 5 before being used to construct a coherent analytic narrative in phase six.

### Reflexive statement

Reflexivity serves as a conscious acknowledgement of the researcher’s assumptions and experiences which influence the research process [40]. This study was conducted by a team of seven medical students alongside our supervisor, each with varying experiences which have shaped our perceptions of primary care. We are aware of our biases towards hierarchy in healthcare teams. However, to reduce the influence of preconceived biases we used open questions to allow free expression and had three researchers conduct the interviews to ensure triangulation.

## Results

This study explored the facilitators and barriers of psychological safety in the four primary care teams. The data analysis yielded three meta-themes: Barriers to psychological safety, facilitators of psychological safety, and shared beliefs.

**Fig. 1:**
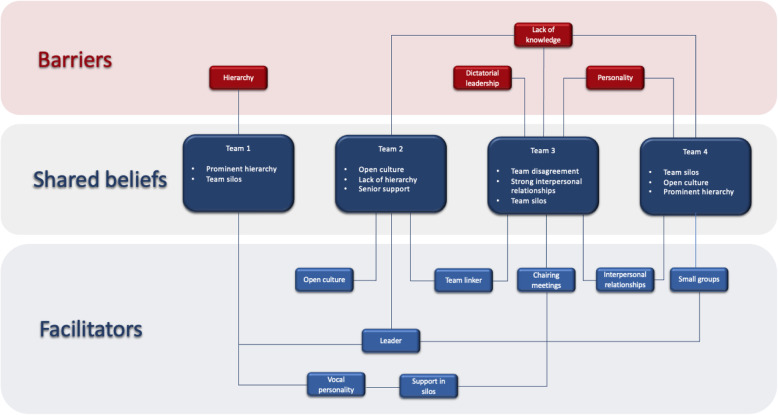
Illustration of primary care teams with their respective shared beliefs, alongside the barriers and facilitators to psychological safety. Lines connecting barriers and facilitators to shared beliefs indicate contextual relation

Facilitators and barriers of psychological safety are the main focus of this study, however, the additional meta-theme of shared beliefs was found to be significantly distinct from barriers and facilitators. Notably, the meta-theme shared beliefs refers to the characteristics of the team, including team dynamics and relationships, and hence provides a common basis for the interpretation of how the facilitators and barriers of psychological safety influence the respective primary care team. Figure 1 summarises the shared beliefs across the four primary care teams, as well as their relation to barriers and facilitators of psychological safety.

### Barriers

The four barriers (hierarchy, lack of knowledge, authoritarian leadership, personality) identified in this study were categorised as either organisational, team-based or individual-level barriers. An overview of the barriers and supporting quotes are shown in Table 2.

**Table 2:** Barriers to psychological safety identified in this study

Barrier		Quote	Level
Hierarchy	MDT members such as doctors valued more	*“Sometimes we can feel the kind of separation like you feel like your input is slightly valued less than a doctor’s would be”*	Organisational
		*“We have had a few incidences where doctors can talk down to us as if it’s as though we aren’t as knowledged as them(…)so it can be a bit soul-destroying sometimes”*	
Lack of Knowledge	Lack of awareness of the cases being discussed	*“because of working (part-time) (...) I probably don’t know enough about that particular subject so I won’t speak but it frustrates me sometimes because I’d like to but I probably wouldn’t in case I’m saying the wrong thing”*	Team based
	Increased anxiety related to saying something incorrect or appearing as the lone member lacking in knowledge	*“I realised it was very clear for the rest of the team but me as for what action has to be taken clinically. So I kind of wrapped up the discussion because I realised there were a few things I didn’t think of that were obvious for the rest”*	
Authoritarian Leadership	Discussion’s being ‘imposed’ rather than being discussed Member’s feeling powerless in clinical decision making	When the leadership *“is not really nice or [is] authoritative or rude, then you know, there’s not much [they] can do because eventually it’s their practice”*	Team based
	Leaders devaluing ideas by team members	*“You need an essential body of leadership to listen, identify and act and we don’t have that. There’s loads of people with good ideas on the ground for the practice but it doesn’t relate to sensible decisions higher up because it’s kind of a vacuum of leadership in the centre of the organization.”*	
Personality	Dominant personalities overpowering conversations Other members unable to contribute	*“There’s quite a mix of personalities and dynamics within the group(...)sometimes just trying to get your point across, so you might have to bring it up several times and you might have to repeat yourself a few times”* *“Sometimes one of the partners might have been a bit more dominant in their opinion and not everybody liked it* “	Individual level
	Intrinsic barriers: shy personality, lack of confidence, fear of public speaking & personal worries about self-image	*“I think I’ve got that- the problem with me is feeling embarrassed, that’s my problem. I don’t think it’s anything to do with the team … they’ve never made me feel stupid”*	

Hierarchy was identified as an organisational level barrier to psychological safety within team 1. This fostered feelings of inferiority and a perception that other members valued their opinions less, increasing hesitancy to voice opinions. Team-based barriers included a lack of knowledge (team 2, 3 and 4) and authoritarian leadership (team 3). The perceived lack of knowledge was attributed to a lack of awareness around the respective discussion topic. This subsequently increased anxiety related to saying something incorrect or appearing as the lone member lacking in knowledge. Furthermore, authoritarian leadership hindered psychological safety with individuals feeling that decisions were enforced rather than discussed. This fostered a lack of ownership and members feeling powerless. Frustrations were two-fold: some participants were discouraged at the domineering approach to decision making, while others expressed concerns over the decisions made.

On an individual level, personality was cited as a barrier to psychological safety. Dominating personalities, particularly of those in leadership roles, acted as a barrier to psychological safety in Teams 3 and 4, by causing unequal dynamics and participation within conversations. Members also expressed that their opinions had to be repeated multiple times to be heard. Furthermore, one team member discussed intrinsic barriers such as shy personality or a fear of public speaking.

### Facilitators

The eight key facilitators (leaders and leader inclusiveness, open culture, support in silos, boundary spanner, interpersonal relationships, small groups, vocal personality, chairing meetings) identified in this study were categorised as either team-based or individual-level barriers. An overview of the facilitators and supporting quotes are shown in Table 3.

**Table 3:** Facilitators of psychological safety identified in this study

Facilitator		Quote	Level
Leader and leader inclusiveness	Introducing individuals to the team	*“The manager makes it a point that they will introduce everybody to the new person... so that you’re not sitting there feeling like nobody knows who you are and you’re not really allowed to say anything”*	Team-based
	Leader actions and qualities, such as active encouragement of participation in MDT discussion, supportive nature and effective listening skills	*“The senior clinician asks every single person if there are any issues, if there is something else to discuss, if they are having any problems”*	
Open culture	Non-judgemental atmosphere	*“Everybody can speak up (...)especially when the nurses and healthcare assistants, they’re all chipping in as well, you do feel very much like I can say whatever want and (...)it’s quite a safe environment as well because nobody judges you”*	Team-based
	Receptiveness to contributions from all members	*“Sometimes you might not get an idea, and a simple layman person may give you an idea that works. And people accept it, they appreciate it and that’s why it is easy for us to communicate”*	
Support in silos	Identifying with a group of similar individuals (a silo) strengthened their voice and created unity within the subgroup.	*“In the nursing team, we’ve all learnt how to stand our ground a bit more that also quite important otherwise it’s a challenge because if a doctor asks you to do something the kind of traditional idea is that they are in authority so it can be difficult to push back”*	Team-based
	A silo leader reduced the power distance by acting as a spokesperson for the group.	*“If something happened it’s easier for me personally to discuss and explain with my head of nurse than going to the manager or the partners which might be easy for my head of nursing team to explain it further and ask for a solution”*	
Vocal personality	Having an inherent trait that enables an individual to voice opinions confidently.		Individual-level
Boundary spanner	The presence of a boundary spanner, an individual responsible for linking sub-groups within the wider MDT, often identified as the practice manager.	This individual was described as essential in ensuring *“a link between admin and clinical teams”*.	Team-based
Chairing meetings	Chairing meetings facilitated individuals to speak up, and in turn, they acted as a facilitator to others speaking up	*“I’m the chair of the meeting so I feel comfortable to express myself.”* *“I’ve also chaired lots of meetings as well so I am aware of the need to get everyone, to encourage everyone’s contribution”*	Individual- level
Interpersonal relationships	Longstanding members with stronger interpersonal relationships felt more comfortable speaking up compared to new individuals to the team.	*“I’ve worked at the practice for five years so I know everybody very well and we’re all very comfortable in speaking our mind. I think when I first started at the practice, I was probably a little bit more hesitant to say my opinions.”*	Team-based
Small teams	Small teams help individuals to be more comfortable and confident, whilst preventing individuals feeling outnumbered	*“I might say it later in a smaller group of um, of GPs and/or nurses but probably not in- in the bigger group.”*	Team-based

Leaders (teams 1,2 and 4) were cited as a prominent facilitator of psychological safety. Within team 1 and 2, leaders exhibiting a friendly attitude, acting in a supportive manner and inviting participation of members made them influential in facilitating psychological safety. An interesting facilitator of psychological safety which surfaced was that of groups of similar individuals in the same profession; silos (teams 1 and 3). Here, psychological safety was facilitated via two mechanisms: identifying within the silo which strengthened voice and empowerment via a silo leader, an individual with reduced power distance who acted as a spokesperson for the group. For example, several members felt more comfortable approaching their nursing team leader or a GP colleague rather than practice leadership directly.

The presence of a boundary spanner, an individual responsible for linking sub-groups within the wider MDT, was cited by participants in teams 2 and 3 as an influential facilitator of psychological safety. Fostering strong interpersonal relationships was an important facilitator of psychological safety in team 3 and 4. One member contrasted their ability to speak up as a longstanding team member compared to being a newcomer, highlighting that knowing the team enabled them to speak up. The presence of a smaller group made participants of Team 4 more comfortable and confident in voicing their opinions.

Individual level facilitators were having a vocal personality and chairing meetings. Vocal personality was a prominent facilitator in teams 1 and 3, with members in team 1 acknowledging their inherent confidence allowed them to voice opinions confidently. An interesting facilitator reported in team 3 was chairing meetings. Some participants referred to the dual perspective of the chairing role, describing that it facilitated them to speak up but they, in turn, acted as a facilitator for others.

## Discussion

To the authors’ knowledge, this is the first qualitative team-based study investigating barriers and facilitators of psychological safety in primary care teams. Obtaining the viewpoints of different healthcare professionals across four primary care teams enabled intra- and inter-group analysis, on the background of shared beliefs, which provided a contextual representation of the team dynamic. The themes that surfaced from this study can be considered at three levels; organisation, team and individual levels.

Barriers and facilitators of psychological safety emerged at an individual level, with personality influencing team dynamics significantly. Whilst the literature reporting on healthcare teams highlights how the behaviour and personality of a leader specifically can be a barrier to psychological safety [4, 41–43], the impacts of dominating personalities amongst other team members is less explored. A shy personality was reported as a barrier, and whilst this may be viewed as an innate characteristic, the influence of the team in negating this should be considered. In contrast, a vocal personality emerged as a facilitator of psychological safety in this study. A relationship between personal control and voicing behaviours has been documented in healthcare literature, whereby individuals with greater autonomy feel empowered to speak up [44], however there is less exploration of the impacts of personality on speaking up behaviours in the context of psychological safety. These findings indicate that psychological safety relies on exploring the personality of both oneself and others in a team in order to establish how individuals can be best supported in the work environment.

Furthermore, our results identified barriers and facilitators at the team level. Our findings revealed that leadership roles are influential as facilitators or barriers to psychological safety. Teams 1,2 and 4 highlighted leaders who displayed support and inclusiveness as facilitators of psychological safety. Where leadership was not cited as a facilitator, it surfaced as a barrier in the form of authoritarian leadership. Literature corroborates this, highlighting a correlation between effective or inclusive leadership and psychological safety in healthcare teams [2, 7, 12, 18, 21, 45–47]. In contrast, leader unreceptiveness has been reported as a barrier to raising patient concerns [18, 19]. A key differentiator between the teams is their leadership structure in the GP practice. Members of a mono-leadership referred to their leader centralising control; this phenomenon may not have emerged in teams with multiple GP partners in the leadership structure. Although this authoritarian leadership style presents benefits in certain situations, such as emergencies occurring commonly in secondary care which require fast decision making by a single leader [48],, this is arguably less applicable and useful in primary care. Crucially, high-performing healthcare organisations are associated with broad leadership distributions [49]; our findings suggest that this should be reflected in primary care.

Through this study, various leadership traits emerged as facilitators to psychological safety, offering practical actions that can be adopted going forwards. This includes showing support, actively listening to team members and inclusive behaviours, such as encouraging contributions or introducing new members of the team to their colleagues. Developing these positive leadership traits is an important step for the NHS, with action already demonstrated by the General Practice Forward View (GPFV), which states that a larger proportion of the primary care budget is being allocated towards the leadership development of more senior GPs [50]. These findings are further supported by the literature, which has highlighted the correlation between effective leadership behaviours and psychological safety in healthcare teams [46, 47, 51] Additional traits that should be adopted by healthcare leaders highlighted by literature include transformational leadership behaviours [52], encouraging innovative change [2] and displaying role-modelling behaviours [15, 43, 53, 54].

Associating within a silo enabled members in teams 2 and 3 to speak up. It appears counterintuitive that profession-based silos, often considered destructive to team cohesiveness [55], could facilitate psychological safety. Perhaps individuals find ‘strength in numbers [56] and subsequently leverage their silos to be heard. This appeared to be particularly noted in teams who reported poor leadership and a prominent hierarchy, both of which emerged as barriers to psychological safety. Although we have identified support in silos as a potential facilitator of psychological safety, caution is needed regarding its practical use. It is possible that this emerges within teams lacking psychological safety, resulting in a reliance rather than support within the silos. This is a novel finding, and further research is required to investigate the underlying role of silos in ensuring psychological safety.

As shown by Jain et al [57], our results also demonstrated the importance of a boundary spanner as a facilitator of psychological safety. However, our study builds on existing literature by suggesting that the practice manager, a non-clinical member of a primary care team, is most appropriate for this role. This likely stems from their knowledge of both clinical and non-clinical activities occurring within a GP practice [58]. This was a facilitator common to two highly contrasting teams (teams 2 and 3), built on different underlying shared beliefs. As primary care teams become increasingly diverse [59], our findings therefore call for the designation of a boundary spanner, given their inextricable value for unifying any team regardless of underlying dynamics. Furthermore, given this increasing diversity in healthcare teams, the traditional hierarchical view whereby doctors are seen as ‘automatic leaders’ [60] is outdated. Our findings show that providing individuals with the opportunity to chair meetings can facilitate voicing behaviour amongst members who are typically reluctant to speak up.

Of particular note is the obstructive effects of hierarchy on psychological safety. The hindering nature of hierarchy is supported by literature, and both our study alongside other research highlight that open cultures can help to negate the impact of hierarchy [61]. However, adopting a team view on hierarchy and open cultures is perhaps too restrictive; rather, a broader view which encompasses the entire healthcare organisation is warranted. Hierarchy is a deep-rooted cultural aspect of healthcare, and while some literature suggests that it can improve role clarity and coordination within teams [62], it is becoming apparent that the resulting detriment to teams should be further acknowledged in healthcare [63]. Our study has shed light on the numerous methods by which teams can help to foster psychological safety. However, if the underlying problems surrounding hierarchies are not addressed at the organisational level, it will still be difficult to foster psychological safety. We propose larger organisations such as professional bodies work towards informing key stakeholders - both clinicians and management teams, of the benefits of psychological safety as well as the role of hierarchy as a barrier to implementing this.

An element of hierarchy may also be responsible for perceived lack of knowledge acting as a barrier, where those ‘lower’ in hierarchy status incorrectly assume others in the team possess more important information and consider their own knowledge to be irrelevant to the discussion [64]. These cognitive biases can have detrimental effects to patient safety, where individuals do not raise crucial information resulting in patient harm [65]. Many junior HCPs also struggle to speak up against senior, more experienced colleagues when errors are occurring, due to an assumption of superior knowledge possessed by their supervisors [66]. These findings where a perceived lack of knowledge acts as a barrier to psychological safety are widely supported by existing literature on healthcare teams [43, 51, 67]. This indicates that building the confidence of each individual team member is a fundamental step to increasing psychological safety, with the leader’s role being to validate input and encourage contribution from every individual, regardless of position.

### Limitations

The findings of this study should be considered in the context of several limitations. Firstly, we were unable to recruit every team member from the four primary care teams, and therefore may have missed key viewpoints. Secondly, despite the effectiveness of snowball sampling for recruitment, this method can incur selection biases as participants are recruited upon referral [68]. Finally, this study was conducted during the COVID-19 pandemic where primary care was overstretched resulting in heightened workplace stress and altered team dynamics. These unique circumstances may have altered participants’ opinions of psychological safety within their team, which may have impacted our data.

### Implications for practice

This study offers a unique insight to the specific barriers and facilitators of psychological safety in primary care, identifying tangible changes that can be adopted at the individual, team and organisation level. The importance of psychological safety in healthcare is well established, underpinning the patient care that is provided and holding potential to benefit both healthcare workers and patients alike [7, 69].

### Implications for future research

During this study, common themes arose regarding perceptions of psychological safety in primary care. Profession based differences are reported in literature, however, are often generalised across healthcare [70–72]. A direct focus on profession analysis would provide an important insight to the field of psychological safety. By identifying profession specific attitudes, barriers and facilitators, personalised support can be offered to increase the psychological safety within general practice.

Importantly, many of the underlying barriers to psychological safety appear to be ingrained into the culture of the healthcare system. This would require multifaceted changes to deep-rooted beliefs and systems, with scope for future research to identify the most effective methods to achieve this. Alongside these efforts, the focus should be directed on the new generation of healthcare professionals and students. Psychological safety remains a relatively unknown concept to both healthcare students and educators alike [73]. Further research should explore their experience and perceptions of psychological safety, particularly whilst exposed on clinical placements, and identify methods to equip students with the ability to ensure psychological safety is prominent within their future multidisciplinary teams.

## Conclusion

This qualitative study aimed to identify facilitators and barriers of psychological safety in primary care, considered at the individual, team and organisation levels. Leaders are influential within a team since their behaviours can directly facilitate or act as a barrier to psychological safety. However, our study highlights that the responsibility and influence does not solely lie with the leader. Rather, there are several behaviours the team can engage in to directly facilitate or impede psychological safety. By strengthening interpersonal relationships, encouraging a rotating chairperson for meetings and finding support in silos to reduce power distances, a team can create a positive team culture that ultimately supports psychological safety. It is anticipated that these findings will encourage primary care teams to reflect on their team dynamics and adopt the aforementioned strategies to ensure every member’s voice is heard.

## Supplementary Information

**Additional file 1: Supplementary file A**- Interview Schedule.

## Data Availability

The datasets generated and analysed during the current study are not publicly available but are available from the corresponding author on reasonable request.
