# Supplementary material for: Exploring the barriers and facilitators of psychological safety in primary care teams: a qualitative study
Source: BMC Health Serv Res. 2021 Mar 24;21:269. doi: 10.1186/s12913-021-06232-7 (PMC7988250; doi:10.1186/s12913-021-06232-7)
Supplement: Supplementary file 1 — Additional file 1: Supplementary file A- Interview Schedule. [file 12913_2021_6232_MOESM1_ESM.docx]

**Supplementary file A- Interview Schedule**

**Roles & Responsibilities**

1. What is your role in the primary care team?

2. Who are the members in your primary care team?

3. What is the role of each member of your primary care team?

**Collaboration**

4. Can you talk to us through a typical MDT meeting?

5. Can you tell us about the last time when your team worked well together?

a. What do you think made that possible?

6. Can you tell us about the last time you felt your team didn’t work so well together?

a. What do you think made that possible?

7. What are the biggest challenges in working in the team in caring for patients?

8. What have you found to be effective in overcoming these challenges?

**Hierarchy**

9. Can you describe to us the distribution of responsibility with your team?

a.What do you think of this distribution of responsibility?

10. How would you describe the hierarchy in your team? *What’s your opinion of the* *hierarchy?*

11. Can you describe a situation when having this hierarchy has been beneficial?

12. Can you think of a time where having a hierarchy has been a drawback for your team?

**Communication**

13. Can you think back to the most recent time in a meeting when you wanted to say something, and you didn’t?

a. Can you tell us why?

14. Could you tell us about the last time you disagreed with someone's opinion in the practice?

And can you tell us how you resolved the situation?

**Psychological safety**

**There is a concept in the social sciences called psychological safety, which is defined as a belief that one will not be punished or humiliated for speaking up with ideas, questions, concerns or mistakes.**

16. What do you think facilitates a psychological safe environment in the team?

17. What do you think could be the barriers to having a psychologically safe environment?

18. Which member of the team is most influential in ensuring a psychologically safe environment?

19. Can you think of a situation where you've felt encouraged to speak up in your team?

20. Can you think of a situation where you've felt discouraged to speak up in your team?
